# Spinal Cord Injury Without Radiographic Abnormality Complicated by Acute Cholecystitis: A Case Report Highlighting Diagnostic and Therapeutic Challenges

**DOI:** 10.7759/cureus.104032

**Published:** 2026-02-21

**Authors:** Yuya Saeki, Yoshihisa Fujinami, Keiji Sato, Manabu Kirita

**Affiliations:** 1 Department of Emergency Medicine, Kakogawa Central City Hospital, Kakogawa, JPN

**Keywords:** acute abdomen, cholecystitis, emergency diagnostics, murphy's sign, spinal cord injury

## Abstract

A man in his early 90s was brought to our emergency department after a fall in which he struck his left shoulder and subsequently became unable to move his left upper extremity. On arrival, neurological examination revealed muscle weakness and paresthesia predominantly affecting the left upper and lower extremities below the C6 level. Cervical magnetic resonance imaging demonstrated narrowing of the spinal canal at the C4/5 and C5/6 levels, corresponding to the level of neurological deficits, along with multilevel left-sided foraminal stenosis, leading to a diagnosis of spinal cord injury without radiographic abnormality (SCIWORA).

Laboratory tests showed no evidence of trauma-related coagulopathy or anemia; however, inflammatory markers were markedly elevated (C-reactive protein, 18.87 mg/dL), and cholestatic enzymes were increased (alkaline phosphatase, 158 U/L; γ-glutamyl transpeptidase, 232 U/L). Abdominal computed tomography revealed gallbladder distension with increased pericholecystic fat attenuation and a 6-mm gallstone at the gallbladder neck. Although the patient had no abdominal symptoms and Murphy's sign was negative, acute cholecystitis was suspected based on laboratory and imaging findings.

The inflammatory response initially improved with fasting and antibiotic therapy but worsened again on hospital day 15. Based on the clinical course, acute cholecystitis was definitively diagnosed, and percutaneous transhepatic gallbladder drainage was performed, resulting in the resolution of both cholecystitis and systemic inflammation. In patients with spinal cord injury, physical findings such as Murphy's sign may be absent; therefore, serial and comprehensive assessment incorporating physical examination, laboratory data, and imaging findings is essential for accurate diagnosis and appropriate management.

## Introduction

Spinal cord injury without radiographic abnormality (SCIWORA) accounts for approximately 10-12% of spinal cord injuries in adults and is increasingly recognized in elderly patients with pre-existing cervical spinal canal stenosis due to degenerative spondylosis [1]. SCIWORA is classically defined as the presence of objective clinical signs of spinal cord injury without evidence of vertebral fracture or malalignment on plain radiographs or computed tomography (CT). In adults, magnetic resonance imaging (MRI) may reveal soft tissue abnormalities such as cord edema, ligamentous injury, or disc herniation despite the absence of osseous injury on CT. Therefore, the diagnosis of SCIWORA relies on neurological deficits in the absence of radiographic evidence of bony trauma, with MRI serving to characterize intramedullary or soft tissue changes rather than to exclude the diagnosis. The term SCIWORA is considered applicable in elderly patients with pre-existing degenerative cervical stenosis; however, spinal cord injury occurring without degenerative stenosis and without CT evidence of trauma is sometimes referred to as spinal cord injury without computed tomography evidence of trauma (SCIWOCTET) [2]. In the present case, pre-existing cervical spinal canal stenosis was identified, and no vertebral fracture or instability was observed on CT imaging. Therefore, the term SCIWORA was considered appropriate, as the neurological deficits occurred in the setting of degenerative canal narrowing without radiographic evidence of bony trauma. Despite the absence of radiographically evident vertebral fractures, patients with SCIWORA may present with a broad spectrum of neurological deficits, including sensory disturbance, motor weakness, abnormal reflexes, and bladder or bowel dysfunction [3]. Because neurological findings may fluctuate or partially recover over time, clinical assessment may be confounded.

Acute cholecystitis, most commonly caused by gallstone obstruction, develops in approximately 1-3% of patients with cholelithiasis annually and is associated with substantial morbidity in elderly individuals [4]. Delayed diagnosis may result in complications such as gangrenous cholecystitis, perforation, sepsis, and increased mortality, particularly in frail patients. Murphy's sign, a classical physical finding in acute cholecystitis, arises from complex interactions between visceral and somatic afferent pathways and is widely used in clinical practice [5]. The sonographic Murphy's sign has been shown to possess greater specificity than the clinical Murphy's sign and may enhance diagnostic confidence in excluding acute cholecystitis; integration of real-time ultrasonographic visualization further augments diagnostic performance [6]. However, even under optimal conditions, its sensitivity remains limited, and a negative finding does not reliably exclude the diagnosis [7].

In patients with spinal cord injury, the reliability and reproducibility of physical examination findings may be compromised due to the disruption of sensory and reflex pathways [8]. Reports specifically addressing the pathophysiological basis for absent Murphy's sign in acute cholecystitis complicated by cervical spinal cord dysfunction remain scarce. Clarifying this diagnostic pitfall has important clinical implications. Herein, we report a case illustrating how reliance on Murphy's sign contributed to diagnostic uncertainty and delayed definitive management of acute cholecystitis in a patient with SCIWORA.

## Case presentation

A 91-year-old man was brought to the emergency department after an accidental fall while attempting to get out of his private vehicle, during which he struck his left shoulder. Following the fall, he was unable to move his left upper extremity, prompting an emergency medical call.

His past medical history included lung adenocarcinoma diagnosed 10 years earlier and managed with observation alone, hypertension, diabetes mellitus, and cataracts. His regular medications consisted of insulin glargine (18 units/day), vildagliptin (50 mg), and amlodipine (5 mg). Prior to admission, the patient was fully independent in activities of daily living. He had a significant smoking history of 40 cigarettes per day for 50 years, having quit at the age of 70 years.

On arrival, the patient's Glasgow Coma Scale (GCS) [9] score was 14/15 (E4V4M6). His vital signs were as follows: blood pressure, 162/70 mmHg; heart rate, 92 beats per minute; respiratory rate, 20 breaths per minute; and oxygen saturation, 94% on room air. Abdominal examination revealed no visible contusions, tenderness, or spontaneous pain.

Neurological assessment using the Manual Muscle Test (MMT) [10] demonstrated a score of 1/5 for left wrist extension and 4/5 for the other upper limb. MMT for hip flexion, knee extension, and ankle dorsiflexion was graded as 2/5 on the right side and 1/5 on the left side. There was no objective sensory loss; however, the patient reported generalized paresthesia involving both upper and lower extremities, accompanied by mild hyperesthesia. In addition, loss of bladder and bowel reflexes was observed (ASIA Impairment Scale [11]: C).

Laboratory investigations performed in the emergency department revealed a marked elevation of C-reactive protein and increased hepatobiliary enzyme levels, including alkaline phosphatase and γ-glutamyl transpeptidase. No evidence of coagulopathy, renal dysfunction, or electrolyte imbalance was identified (Table 1).

**Table 1:** Laboratory tests on admission WBC: white blood cell count; Hgb: hemoglobin; Plt: platelet count; CRP: C-reactive protein; TP: total protein; Alb: albumin; AST: aspartate aminotransferase; ALT: alanine aminotransferase; LDH: lactate dehydrogenase; CK: creatine kinase; T-Bil: total bilirubin; BUN: blood urea nitrogen; Cre: creatinine; Na: sodium; K: potassium; Cl: chloride; eGFR: estimated glomerular filtration rate; Ca: calcium; P: phosphate; Mg: magnesium; Glu: glucose; PT: prothrombin time; APTT: activated partial thromboplastin time; IU: international unit

Parameter	Value (unit)	Reference range (unit)
WBC	6.25 (10^3^/μL)	3.3-8.6 (10^3^/μL)
Hgb	13.7 (g/dL)	11.6-14.8 (g/dL)
Plt	155 (10^3^/μL)	158-348 (10^3^/μL)
CRP	18.87 (mg/dL)	0-0.14 (mg/dL)
TP	6.0 (g/dL)	6.6-8.1 (g/dL)
Alb	3.3 (g/dL)	4.1-5.1 (g/dL)
AST	41 (IU/L)	13-30 (IU/L)
ALT	31 (IU/L)	7-23 (IU/L)
LDH	213 (IU/L)	124-222 (IU/L)
CK	107 (IU/L)	41-153 (IU/L)
T-Bil	1.86 (mg/dL)	0.4-1.5 (mg/dL)
BUN	17.5 (mg/dL)	8-20 (mg/dL)
Cre	0.73 (mg/dL)	0.46-0.79 (mg/dL)
Na	140 (mEq/L)	138-145 (mEq/L)
K	3.3 (mEq/L)	3.6-4.8 (mEq/L)
Cl	104 (mEq/L)	101-108 (mEq/L)
Glu	76 (mg/dL)	73-109 (mg/dL)
HbA1c	6.1 (%)	4.9-6 (%)
PT activity	83.8 (%)	70-130 (%)
APTT	28 (sec)	24-34 (sec)
D-dimer	0.90 (μg/mL)	0-1.0 (μg/mL)

**Figure 1:**
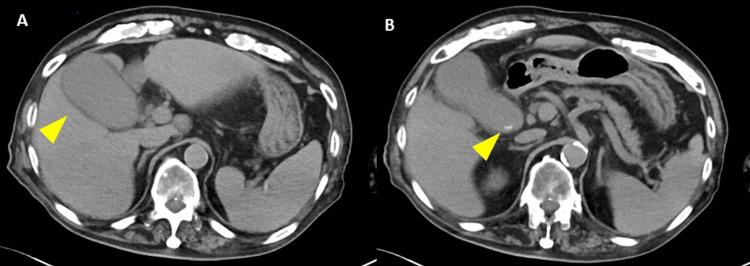
Initial non-contrast abdominal computed tomography (A) Marked distension of the gallbladder with increased density of the surrounding fat tissue. (B) A 6-mm calculus identified at the gallbladder neck.

Ultrasound and CT examination revealed no traumatic abnormalities such as intra-abdominal hemorrhage, free air, or solid organ injury. However, gallbladder distension with increased pericholecystic fat density and a 6-mm gallstone at the gallbladder neck were identified (Figure 1).

**Figure 2:**
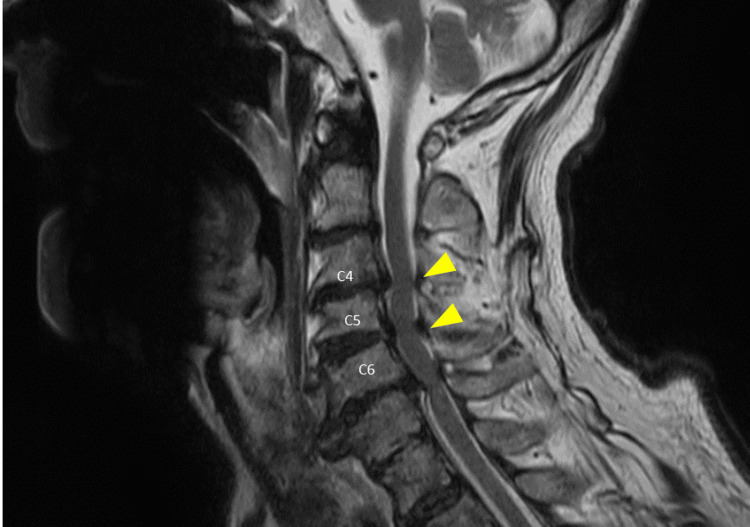
Cervical magnetic resonance imaging on initial evaluation On T2-weighted imaging, no cervical fracture or epidural hematoma was identified, and no definite intramedullary signal abnormality was observed. However, sagittal canal narrowing was noted at the C4/5 and C5/6 levels.

Cervical MRI demonstrated no evidence of cervical vertebral fracture, epidural hematoma, or definite intramedullary signal abnormality. Nevertheless, sagittal narrowing of the spinal canal was observed at the C4/5 and C5/6 levels (Figure 2).

Clinical course

In the emergency department, the patient was diagnosed with SCIWORA and suspected acute cholecystitis. Conservative management with cervical immobilization using a neck collar was initiated for SCIWORA. For the suspected cholecystitis according to Tokyo Guidelines 2018 [7], a surgical consultation was obtained; however, a definitive diagnosis was not established because Murphy's sign remained negative, and empirical antimicrobial therapy with cefotaxime (CTX) was initiated to provide coverage against *Enterobacterales* commonly implicated in biliary tract infections.

**Figure 3:**
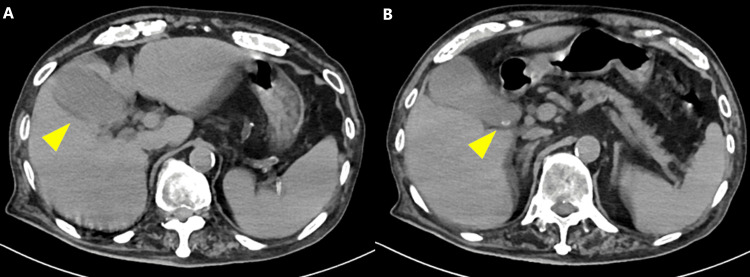
Abdominal non-contrast computed tomography on hospital day 15 (A) Persistent gallbladder distension with increased pericholecystic fat density, showing no significant change from the initial study. (B) A 6-mm gallstone at the gallbladder neck, unchanged from the initial study.

For the conservative management of acute cholecystitis, the duration of antimicrobial therapy was determined to be approximately five days, consistent with the recommendations of the World Society of Emergency Surgery (WSES) guidelines [4]. The patient remained afebrile, and inflammatory markers gradually normalized; therefore, antibiotic therapy was discontinued on hospital day 7. However, inflammatory markers increased again on hospital day 15. Murphy's sign remained negative, and repeat CT demonstrated persistent findings suggestive of acute cholecystitis (Figure 3). Based on these findings, recurrent cholecystitis was definitively diagnosed, and fasting with antibiotic therapy was resumed.

**Figure 4:**
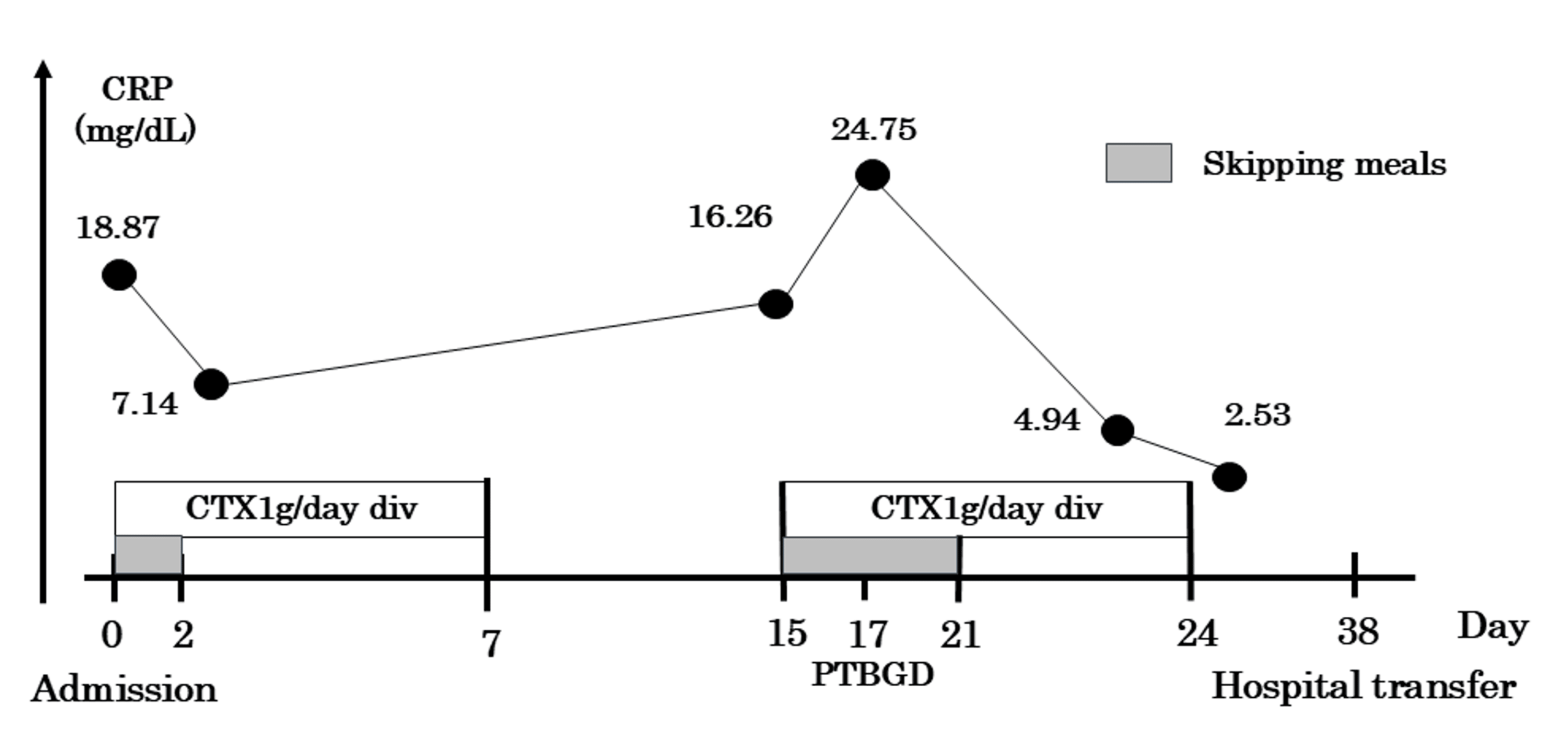
Clinical course Antimicrobial therapy with CTX was initiated upon admission and continued for seven days, resulting in an initial improvement in inflammatory markers. However, on hospital day 15, inflammatory markers were worsened again. Although the possibility of antimicrobial resistance could not be entirely excluded, inadequate treatment duration was considered the more plausible explanation. Therefore, CTX therapy was reinitiated. A PTGBD procedure was performed on hospital day 17. The inflammatory response subsequently showed a sustained downward trend and resolved by hospital day 24. The patient remained clinically stable thereafter and was transferred to a rehabilitation facility on hospital day 38. CRP: C-reactive protein; CTX: cefotaxime; PTGBD: percutaneous transhepatic gallbladder drainage

Given the patient's frailty, surgical intervention was deemed inappropriate by the surgical team. Consequently, percutaneous transhepatic gallbladder drainage was performed by the gastroenterology team on hospital day 17. Following the procedure, the patient's cholecystitis improved, and he was subsequently transferred on hospital day 37 to a rehabilitation facility for the continued management of SCIWORA (Figure 4).

## Discussion

This case describes an incidental cervical spinal cord injury complicating underlying acute cholecystitis, in which definitive diagnosis was challenging because Murphy's sign remained persistently negative. Murphy's sign, defined as the elicitation of pain with abrupt cessation of inspiration during deep inhalation while applying pressure to the right subcostal region, is widely recognized as a characteristic physical finding of acute cholecystitis. However, its diagnostic accuracy varies considerably across studies. A systematic review of emergency department patients reported a sensitivity of approximately 62% and a specificity of approximately 96%, yielding a high positive likelihood ratio (LR+ 15.6), whereas the negative likelihood ratio remained modest (0.40), indicating that a negative Murphy's sign does not exclude acute cholecystitis [5].

The Tokyo Guidelines 2018 include Murphy's sign as part of the diagnostic criteria for acute cholecystitis while explicitly acknowledging its limited sensitivity. Yokoe et al. reported that although the sensitivity of Murphy's sign was generally estimated at 50-60% in earlier studies, a single-center study demonstrated a markedly lower sensitivity of 20.5%, leading to the conclusion that while specificity is high, the diagnostic value of a negative finding is limited [7]. Thus, although the presence of typical physical findings warrants early therapeutic intervention for acute cholecystitis, the absence of such findings should not be used to rule out the disease.

Although there are no reports directly demonstrating that spinal cord injury results in a negative Murphy's sign, numerous studies have documented that patients with spinal cord injury often present with acute abdominal conditions lacking typical physical findings [8]. Pain associated with cholecystitis is primarily perceived through visceral afferent fibers projecting to the spinal cord via the sympathetic nervous system. Visceral afferent pathways are broadly categorized into vagal, sympathetic (thoracolumbar sympathetic chain and splanchnic nerves), and pelvic visceral nerve systems. Afferent fibers from upper abdominal organs, including the gallbladder, travel along thoracic splanchnic nerves, particularly the greater splanchnic nerve, and terminate in the dorsal horn of the upper to mid-thoracic spinal cord (approximately T7-T9), where they converge with somatic afferent fibers, resulting in the perception of right upper quadrant or epigastric pain [12].

On the other hand, Murphy's sign does not represent gallbladder pain per se but rather a reflexive response characterized by pain perceived via diaphragmatic movement and transient cessation of inspiration. This response involves visceral afferent pathways from the gallbladder, liver capsule, and peritoneum (via sympathetic visceral nerves), somatic nerves innervating the abdominal wall and intercostal muscles (intercostal nerves), the phrenic nerve (C3-C5) innervating the diaphragm and its peritoneum, and integrative reflex circuits at the spinal and brainstem levels [13]. In spinal cord injury, disruption of somatic sensation distal to the lesion, particularly abdominal cutaneous sensation derived from thoracic segments, may impair sensory transmission, rendering Murphy's sign absent or difficult to evaluate. Furthermore, injuries distal to the thoracic spinal cord may impair voluntary control of abdominal musculature, thereby interfering with respiratory modulation in response to painful stimuli and contributing to the absence of Murphy's sign.

In the present case, the absence of objective abnormalities on superficial sensory and pain testing may be explained by partial impairment of visceral afferent pathways due to cervical cord edema or spinal canal stenosis, as well as impaired coordination of respiratory and truncal muscles, which are necessary to elicit Murphy's sign through deep inspiration and abdominal wall tension. Additionally, advancing age has been associated with the attenuation of peritoneal irritation signs in acute abdominal conditions as well as reduced positivity of Murphy's sign in acute cholecystitis [14]. Diabetes mellitus has also been reported to blunt abdominal symptoms through autonomic neuropathy-related mechanisms [15]. Accordingly, the patient's extreme age and insulin-dependent diabetes mellitus may have further contributed to the absence of Murphy's sign in this case.

Previous studies have also demonstrated a higher prevalence of cholelithiasis and cholecystitis among patients with spinal cord injury, potentially related to prolonged immobilization, nutritional changes, and autonomic dysfunction [16]. Despite this increased incidence, the lack of typical abdominal symptoms often leads to delayed diagnosis and disease progression. Therefore, in patients with acute abdominal conditions complicated by cervical spinal cord injury, clinicians should not rely solely on physical examination findings. As demonstrated in this case, Murphy's sign may be absent even in the presence of active cholecystitis, underscoring the importance of integrating serial physical examinations with laboratory data and imaging findings to achieve timely and accurate diagnosis.

## Conclusions

We report a case in which reliance on classical physical findings contributed to the delayed confirmation of acute cholecystitis during the conservative management of SCIWORA. In patients with spinal cord injury, clinicians should not exclude acute cholecystitis solely on the basis of a negative Murphy's sign or absence of abdominal symptoms. Instead, early imaging evaluation and close monitoring of clinical and inflammatory trends should be performed when biliary pathology is suspected, particularly in elderly or neurologically impaired patients. Recognizing this diagnostic pitfall may help prevent delayed intervention and subsequent complications.
